# Metabolic Profiling and Gene Expression Analysis Unveil Differences in Flavonoid and Lipid Metabolisms Between ‘Huapi’ Kumquat (*Fortunella crassifolia* Swingle) and Its Wild Type

**DOI:** 10.3389/fpls.2021.759968

**Published:** 2021-12-02

**Authors:** Qiaoli Ma, Yongwei Hu, Xinghua Dong, Gaofeng Zhou, Xiao Liu, Qingqing Gu, Qingjiang Wei

**Affiliations:** ^1^College of Agronomy, Jiangxi Agricultural University, Nanchang, China; ^2^National Navel Orange Engineering Research Center, Gannan Normal University, Ganzhou, China; ^3^School of Horticulture and Plant Protection, Yangzhou University, Yangzhou, China

**Keywords:** metabolomics, flavonoids, lipids, kumquat fruit, gene expression

## Abstract

To elucidate the mechanism underlying special characteristic differences between a spontaneous seedling mutant ‘Huapi’ kumquat (HP) and its wild-type ‘Rongan’ kumquat (RA), the fruit quality, metabolic profiles, and gene expressions of the peel and flesh were comprehensively analyzed. Compared with RA, HP fruit has distinctive phenotypes such as glossy peel, light color, and few amounts of oil glands. Interestingly, HP also accumulated higher flavonoid (approximately 4.1-fold changes) than RA. Based on metabolomics analysis, we identified 201 differential compounds, including 65 flavonoids and 37 lipids. Most of the differential flavonoids were glycosylated by hexoside and accumulated higher contents in the peel but lower in the flesh of HP than those of RA fruit. For differential lipids, most of them belonged to lysophosphatidycholines (LysoPCs) and lysophosphatidylethanolamines (LysoPEs) and exhibited low abundance in both peel and flesh of HP fruit. In addition, structural genes associated with the flavonoid and lipid pathways were differentially regulated between the two kumquat varieties. Gene expression analysis also revealed the significant roles of *UDP-glycosyltransferase* (*UGT*) and *phospholipase* genes in flavonoid and glycerophospholipid metabolisms, respectively. These findings provide valuable information for interpreting the mutation mechanism of HP kumquat.

## Introduction

Kumquat is of the genus *Fortunella* that is closely related to *Citrus* genus. Both belong to the true *Citrus* group of the Rutaceae family. As an important part of the most popular fruit crops of citrus, kumquat is widely distributed and cultivated in the world, particularly in southeast China. It is a favorite by consumers due to its good taste, small size, and being eaten raw without peeling. Besides its attractive organoleptic properties, kumquat is an excellent source of nutrients and phytochemicals, such as flavonoids, phenolic acids, carotenoids, vitamins, polysaccharides, essential oil, lipids, and dietary fiber (Liu et al., 2019). Furthermore, it is an important herbal medicine in Chinese traditional medicine. Its dry roots, leaves, and fruits even have been commonly employed to treat traumatic injury, colds, and coughs (Rafiq et al., 2018). And in modern medicine, some bioactive compounds have been isolated from ripening or unripe fruit to study their pharmacological activities in immune defense, antioxidation, antimetabolic disorder, diabetes, cardiovascular diseases, psychiatric disorder, and cancer (Erlund, 2004; Aruoma et al., 2012). Hence, revealing the constitution and biosynthesis of functional compounds in kumquat fruit will benefit for citrus breeding to produce new resources of functional fruit.

Recently, more attentions have been paid to characterize the natural chemical profiles of citrus fruits (Wang et al., 2017). Flavonoids are a massive class of secondary metabolites in plants and are especially rich in citrus fruits with a more amount of total polyphenol and flavonoid than that of total carotenoids (Tan et al., 2016; Lou and Ho, 2017). Flavonoids are the part of fruit pigmentation and make fruit exhibit special taste, for instance, hesperidin, nobiletin, naringin, tangeretin, and neohesperidin for bitter taste in citrus, and dihydrochalcone for sweet taste (Ververidis et al., 2007). Based on differences in their chemical structures, flavonoids are mainly classified into several basic types, such as flavone, flavonol, flavanone, flavanonol, isoflavone, and isoflavanone (Ververidis et al., 2007). In citrus fruits, flavonoids are usually modified by different chemical groups, including *C*-glycosyl, *O*-glycosyl, -OCH_3_, -OH, and -CH_3_ (Zhang et al., 2012; Khan et al., 2014). And the flavonoids composition in flavedo, albedo, and juice vesicle tissues also showed a large-magnitude variation (Zhang et al., 2011), for example, polymethoxylated flavonoids (PMFs) preferentially accumulated in the flavedo (Wang et al., 2017).

However, little is known about total natural metabolites in kumquat fruits. Barreca et al. (2011) identified and quantified only 13 *C*- and *O*-glycosyl flavonoids in crude juice from kumquat fruits and first reported tvicenin-2, lucenin-2 4′-methyl ether, narirutin 4′-O-glucoside, acacetin 3,6-di-*C*-glucoside, and apigenin 8-*C*-neohesperidoside existing in kumquat juice. Tan et al. (2016) identified 12 compounds in ethanol extract of the whole kumquat fruit by using UPLC Q-TOF/MS, and five of these compounds were tentatively characterized in the four major cultivated *Fortunella* types with 3′,5′-di-*C*-glucopyranosylphloretin, acacetin 8-*C*-neohesperidoside, and acacetin 6-*C*-neohesperidoside as the dominant components. Lou and Ho (2017) reviewed all the flavonoids identified in kumquat and listed the 15 major flavonoids. Overall, the flavonoid profiles in kumquat fruit are limited till now. It is worth noting that based on HPLC-DAD-ESI-MA/MS, Wang et al. (2017) had identified 117 flavonoids in 62 *Citrus* germplasms from five citrus species, namely, sweet orange, mandarins, lemons, pummelos, and grapefruits, but excluded kumquat. This study guided us to identify the flavonoids existing in kumquat fruit.

Besides flavonoids, lipids are essential components of the edible part in kumquat fruits, which play a pivotal role in membrane structure and stability, time of storage, and organoleptic properties of the juices. There were 0.86 g lipids per 100 g edible portion of fresh kumquat, and phospholipids accounted for about 8–18% of the total lipids in different parts of citrus fruits (Kolesnik et al., 1989). Lysophosphatidylethanolamines (LysoPEs) and lysophosphatidycholines (LysoPCs) were the hydrolytic products of glycerophospholipids by PLA2 (Cowan, 2006), and they participated in regulating fruit quality formation and postharvest senescence (Amaro and Almeida, 2013). By using GC, Touns et al. (2011) had analyzed the lipid composition (mainly about fatty acids) of the oilseed in bitter orange and mandarin. But there were few reports of lipids profile in kumquat fruits to our limited knowledge. For the complex and diverse metabolites in citrus fruits, natural product identified in kumquat is very insignificant. Hence, using the existing advanced metabolomics technology, more kumquat metabolites can be isolated and identified, which may further enrich the product data resources of citrus fruits.

‘Rongan’ kumquat (*Fortunella crassifolia* Swingle, RA) is the main variety and has a long cultivation history in the Guangxi Zhuang Autonomous Region, China. ‘Huapi’ kumquat (HP), screened in the 1980s, is a spontaneous seedling mutant that originated from RA kumquat. Compared with the wild type, the mutant HP fruit has attractive traits such as glossy peel, fewer seeds, and less spicy flavor. We previously found that the contents of organic acid and soluble sugar and the expression levels of related genes exhibited large differences between HP and RA fruits (Wei et al., 2021). But the chemical material compositions associated with the morphological and quality differences of the two varieties are not fully understood. To illustrate the biochemical basis for its special traits, metabolic profiling of the peel and flesh derived from HP and RA fruits at the maturation stage was investigated based on a widely targeted metabolomics approach, and the expression patterns of genes related to compound differences were also comprehensively analyzed.

## Materials and Methods

### Plant Materials

Fruits of RA and HP were harvested from 13-year-old trees growing on the same orchard (Rong’an County, Guangxi Zhuang Autonomous Region, China) under similar management conditions. For each variety, fruit samples were collected randomly from the peripheral canopy of at least three trees, with 40 representative fruits from each tree at 210 days after flowering (DAF). After collecting, the fruits were quickly transported to the laboratory in an ice-box. The peel and flesh were separated from each fruit, immediately frozen in liquid nitrogen, and stored at −80°C for further use.

### Analysis of Phenotypic Characteristics and Determination of Flavonoids

Fresh coloration of fruit was determined by a CR-400 chromameter (Minolta, Japan) and described by the indexes of lightness (L*), green-red color value (a*), and blue-yellow value (b*). The citrus color index (CCI), a comprehensive indicator for color impression, was calculated as CCI = 1000 × a*/(L* × b*), and it represents a degree of yellow/orange/reddish of fruit color. Ten fruits were used per replicate with three replicates for each variety. Two measurements were made at two symmetrical points at the equatorial region of each fruit.

Observation and calculation of oil morphology were performed using a light microscope (Olympus IX73, Tokyo, Japan). The fruit peel for scanning electron microscopy (SEM) was prepared as described by Liu et al. (2015). Then, the samples were observed and photographed under a scanning electron microscope (JSM-6390LV, JEOL, Japan). The total content of flavonoids was measured using the plant flavonoids test kit (Nanjing Jiancheng Bioengineering Research Institute, China). The flavonoid content was calculated by reading the absorbance at 502 nm using a spectrophotometer (Shimadzu UV-1800, Japan).

### Metabolic Profiling

The peel and flesh samples acquired above were first freeze-dried and crushed using a mixer mill (MM 400, Retsch). Metabolite identification was carried out using a widely targeted metabolomics method by MetWare Biotechnology Co., Ltd (Wuhan, China) following their standard procedures. Three biological replicates were independently analyzed for each sample. To validate the technical reproducibility, the mixture of the sample extracts was serviced as a quality control sample and was added in the analytical pipeline. In total, 15 datasets, namely, three mixed quality control data (Mix01, 02, and 03) and 12 experimental sample data RA-Peel (RAP1, 2, and 3), RA-Flesh (RAF1, 2, and 3), HP-Peel (HPP1, 2, and 3), and HP-Flesh (HPF1, 2, and 3), were obtained. Mass spectrum signals were qualified and quantified according to the MetWare local database. Quantification of metabolites was carried out using a multiple reaction monitoring (MRM) method. Metabolites with a threshold of log2 (fold change) ≥ 2 or ≤ 0.5 and variable importance in project (VIP) ≥ 1 were considered as differential accumulation. Then, the differentially accumulated metabolites were mapped to the Kyoto Encyclopedia of Genes and Genomes (KEGG) database and used in the significant enrichment analysis and major enriched pathways. The differential metabolites were hierarchically clustered under the algorithm of Euclidean distance and complete linkage by MEV software (v 4.9.0).

### Differential Gene Expression Analysis

To investigate the gene expression profiles, transcriptomic data generated from the peel and flesh of RA and HP fruits from 90, 150, and 210 DAF were downloaded from the NCBI Sequence Read Archive (SRA) database under the accession PRJNA658060 (Wei et al., 2021). Gene expression levels were calculated based on FPKM (fragments per kilobase of exon model per million mapped reads) using RSEM software. Significant difference thresholds [FDR (false discovery rate) ≤ 0.01 and | log2 (fold change)| ≥ 1] were used to screen the differentially expressed genes (DEGs).

### Statistical Analysis

All data were performed using SPSS Statistics (Chicago, IL, United States) and expressed as the mean ± standard error of triplicates. The differences between the means were evaluated using Student’s *t-*test.

## Results

### Fruit Characteristics of RA and HP Kumquats

**FIGURE 1:**
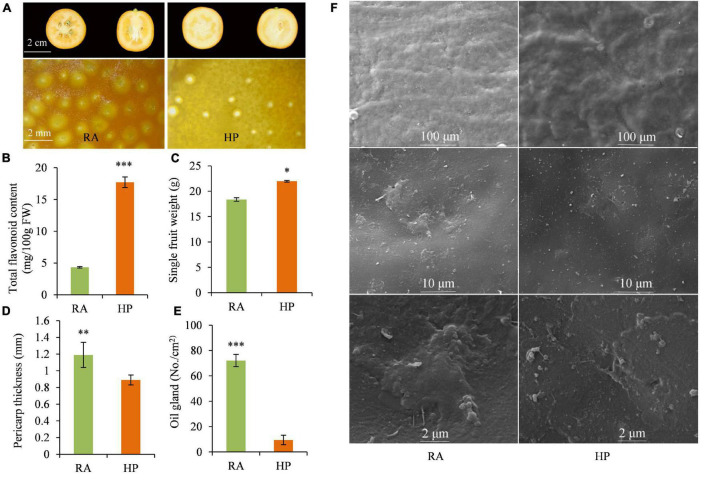
Quality characteristics of RA and HP fruits. **(A)** Fruit photograph. **(B)** Total flavonoid content. **(C)** Single fruit weight. **(D)** Pericarp thickness. **(E)** Oil gland number. **(F)** Cuticle microscopic morphology. Asterisks indicate the significant difference between two verities (*P < 0.05, **P < 0.01, and ***P < 0.001).

Dynamic changes in fruit color of RA and HP kumquats were investigated during fruit development and maturation. Color indexes, including L*, a*, b*, and CCI, showed no difference between HP and RA during the development stage of 60∼150 DAF, but were significantly lower in HP compared to RA during the turning and maturation stage (150–180 DAF) (Supplementary Figure 1). The lower value of CCI in HP indicates a lighter orange/reddish external peel color than in RA fruit. This was in accordance with the color appearance of matured HP (yellow-orange) and RA (reddish-orange) fruit (Figure 1A). As an important type of flavor and pigments component, the total flavonoid content was also detected and compared between HP and RA. There was 17.71 mg/100 g^–1^ FW of flavonoids in HP, which was four times higher than that of RA fruit (4.33 mg/100 g^–1^ FW) (Figure 1B). Furthermore, HP fruit was seedless (Figure 1A) and has a higher single fruit weight (Figure 1C), lower pericarp thickness (Figure 1D), and fewer oil glands in the pericarp (Figure 1E), as well as thinner wax and cuticle (Figure 1F) than those of RA fruit. All these advanced characteristics make HP fruit more glossy and attractive than RA fruit.

### Analysis of the Metabolomics Data Quality and Comparison of Metabolites Between Two Kumquat Varieties

**FIGURE 2:**
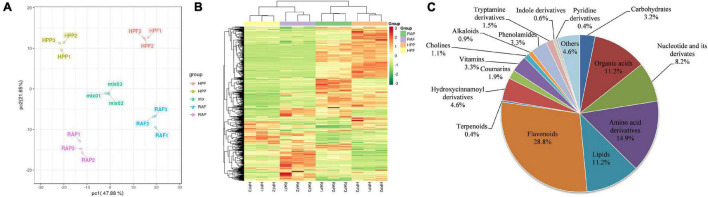
Cluster analysis for all sample data. **(A)** Principal component analysis of the peel and flesh of two kumquat fruits and the mixed samples. **(B)** Hierarchical cluster analysis of all metabolites detected in the peel and flesh of RA and HP samples. Content of a metabolite was normalized by the range method. Each example is visualized in a single column, and each metabolite is represented by a single row. **(C)** Categories and proportion of all identified metabolites.

To comprehensively investigate the biochemical mechanisms of the morphological differences between HP and RA kumquat, all metabolites in the peel and flesh were measured by LC-ESI-MS/MS system. The total ions current (TIC) and multi-peak detection plot (XIC) of metabolites in the MRM mode of a mixed-quality control samples are shown in Supplementary Figure 2. To further understand the overall metabolic variance among those samples, a PCA over the metabolic profiles of the 15 samples was conducted. The first two components of the PCA score plot represent 69.53% of the total variance: The explained values of PC1 and PC2 were 47.88% and 21.65%, respectively. In the PCA plot, any three replicated samples within a group were tightly clustered together, and samples from different varieties or tissues were separated from each other (Figure 2A). These PCA results indicated that mass data were good in repetition, and metabolites in different groups differed distinctively. Hierarchical cluster analysis of all detected metabolites in the 12 experimental samples showed that the three biological replicates of each group clustered together, which further confirmed the high reliability of these generated metabolite data (Figure 2B). Moreover, the two flesh groups of HP and RA were separated from the two peel groups, which was coordinated with the distribution of these four groups on PC1 of PCA.

Mass spectrum signals were qualified and quantified based on the local metabolite database. In total, 538 metabolites were identified in the two kumquat fruits (Supplementary Table 1). Carbohydrates (3.2%), organic acids (11.2%), amino acids, and lipid-related substances (14.9% and 11.2%) in primary metabolites, as well as flavonoids (28.8%) and terpenoids (0.4%), and hydroxycinnamoyl derivatives (4.6%) in secondary metabolites, accounted for 66% of the detected metabolites. It is obvious that flavonoids are the most abundant components and account for about one-third of the total detected metabolites in matured kumquat fruits (Figure 2C).

**FIGURE 3:**
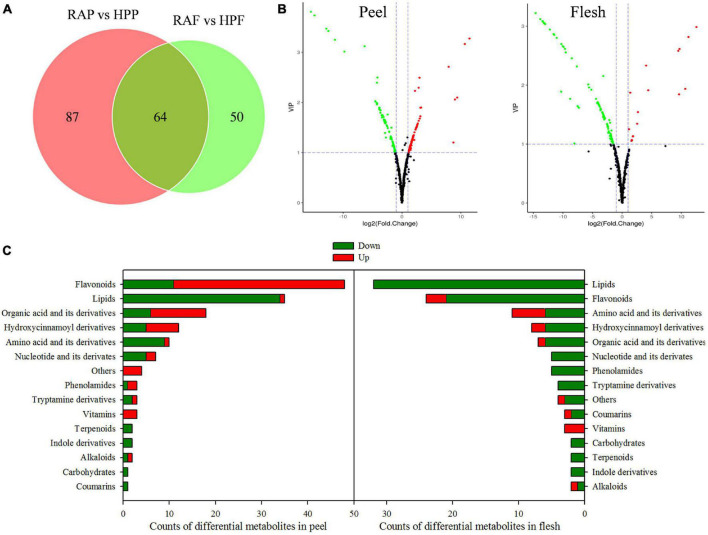
Analysis of the differential metabolites in the peel and flesh of HP compared with RA. **(A)** Venn diagram of the differential metabolites. **(B)** Volcano plot of differential metabolites in the peel and flesh samples, respectively. Red scatter point indicates upregulated metabolite, whereas green scatter point indicates downregulated metabolite. **(C)** Functional categories and number of the differential metabolites.

There were 201 of the 538 metabolites exhibiting significantly different levels between the two kumquat varieties, with 151 metabolites differentially accumulating in the peel and 114 in the flesh, and 64 overlappings between the two tissues (Figure 3A and Supplementary Table 2). Among those differential metabolites, 71 metabolites were upregulated and 80 downregulated in the peel of HP compared to that of RA (Figure 3B). In the flesh, however, most of the differential metabolites (97 out of 114) were downregulated and only 17 metabolites were upregulated in HP (Figure 3B). According to the molecular structure and function, the differential metabolites were categorized into 15 categories (Figure 3C). In the peel, the top four categories were flavonoids (48), lipids (35), organic acids and their derivatives (18), and hydroxycinnamoyl derivatives (12). Similarly, lipids (32), flavonoids (24), amino acids and their derivatives (11), and hydroxycinnamoyl derivatives (8) were also on the top four categories in the flesh of two kumquat fruits (Figure 3C).

### Kyoto Encyclopedia of Genes and Genomes Classification and Enrichment of the Differentially Accumulated Metabolites

To further identify the important metabolic pathways, KEGG classification analysis was performed. Of the 151 differential metabolites in HP peel, 58 were mapped into 62 KEGG pathways that were mainly responsible for the secondary metabolite biosynthesis pathways, such as flavonoid, phenylpropanoids, and alkaloids, and tryptophan metabolism (Supplementary Figure 3A). And of the 114 differential metabolites in HP flesh, 48 were mapped into 56 pathways that mainly were phenylpropanoids, flavonoid, tryptophan, and pyrimidine biosynthesis pathways (Supplementary Figure 3B). Enrichment analysis was further conducted to evaluate the importance of these pathways, and the top 20 pathways are shown in Supplementary Figure 3. There were five pathways significantly enriched in the peel, namely, (1) phenylalanine, tyrosine, and tryptophan biosynthesis; (2) stilbenoid, diarylheptanoid, and gingerol biosynthesis; (3) tryptophan metabolism; (4) staurosporine biosynthesis; and (5) anthocyanin biosynthesis (Supplementary Figure 3C). However, only the tryptophan metabolism pathway was significantly enriched in the flesh (Supplementary Figure 3D).

### Differences of Flavonoid and Lipid Accumulation Between HP and RA Kumquats

**FIGURE 4:**
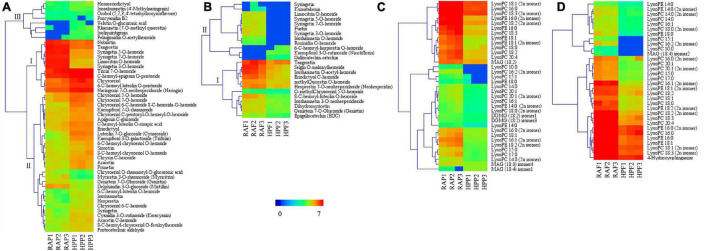
Heat map visualization of the relative differences in flavonoids and lipids in the peel and flesh of HP compared with those of RA. **(A)** Flavonoids in the peel and **(B)** in the flesh. **(C)** Lipids in the peel and **(D)** in the flesh. The content of each metabolite was logarithm-normalized to complete linkage hierarchical clustering. Each column represents one sample, and each single row represents one metabolite. Red to dark blue indicates abundance changes from high to low.

Considering the wide variations of flavonoids and lipids in both peel and flesh in the two kumquat fruits, we conducted a detailed analysis of these two compounds. In the peel, most of the differential flavonoids were glycosylated by hexoside, and the glycosides often occurred at 3-*O*, 5-*O*, 7-*O*, 6-*C*, and 8-*C*. Compared with RA, 37 out of the 48 flavonoids were upregulated in HP kumquat. According to the accumulation and change mode, the 48 differential flavonoids were classified into three clusters (Figure 4A). Metabolites in cluster I were the most abundant substances in the peel and their variation accounted for 79% of the total difference between HP and RA fruits. Among these metabolites, tricin 7-*O*-hexoside, *C*-hexosyl-apigenin *O*-pentoside, naringenin 7-*O*-neohesperidoside, chrysoeriol, and 6-*C*-hexosyl luteolin *O*-pentoside were highly accumulated in HP, whereas limocitrin *O*-hexoside, syringetin 3-*O*-hexoside, syringetin 7-*O*-hexoside, syringetin 5-*O*-hexoside, tangeretin, and nobiletin were highly accumulated in RA. There were 29 metabolites in cluster II, which both moderately accumulated in the peel and showed higher accumulation in HP. Eight metabolites in cluster III with trace accumulation in the peel were relatively highly accumulated in HP; among them, velutin *O*-glucuronic acid and isoliquiritigenin were only detected in HP. In contrast with the peel, most of these flavonoids (21 out of 24) were downregulated in the flesh of HP kumquat (Figure 4B). And they were clustered into two classes. Metabolites in cluster I were abundantly accumulated in both kumquat fruits: Tangeretin, selgin *O*-malonyl hexoside, isorhamnetin *O*-acetyl-hexoside, eriodictyol *C*-hexoside, neohesperidin, and methyl quercetin *O*-hexoside were particularly lower in HP than in RA. Metabolites in cluster II were tiny accumulated either in HP or in RA. Furthermore, only seven differential flavonoids overlapped between the peel and the flesh, namely, genistein 7-*O*-glucoside, limocitrin *O*-hexoside, syringetin, syringetin 3-*O*-hexoside, syringetin 5-*O*-hexoside, syringetin 7-*O*-hexoside, and tangeretin.

Lipids are an important constituent of plant cells. Almost all differential lipids were less in amount in HP than in RA (Figures 4C,D), and their distinction between the two kumquats was very similar among different tissues; 30 of 37 differential lipids were overlapped. The overlapped differential lipids belonged to either LysoPCs or LysoPEs, which were the hydrolytic products of glycerophospholipids catalyzed by several phospholipases. Furthermore, LysoPC 18:3, 18:2, 18:1, and 16:0 and LysoPE 18:1 and 16:0 are the main differential lipids between HP and RA fruits.

### Differential Expression of Genes Related to Flavonoid and Lipid Metabolism

**FIGURE 5:**
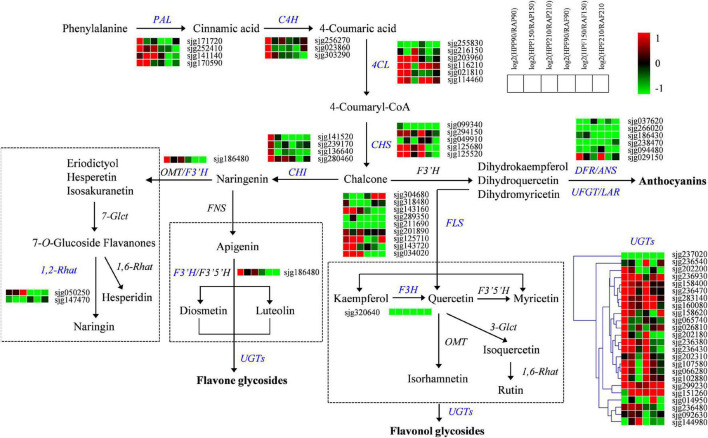
Expression profiles of genes from the flavonoid biosynthesis pathway in the peel and flesh of kumquat fruits. The color scale from green (low) to red (high) represents the values of the log2 ratio (HP/RA) at 90, 150, and 210 DAF. The differentially expressed genes are highlighted in blue. PAL, phenylalanine ammonia-lyase; C4H, trans-cinnamate 4-monooxygenase; 4CL, 4-coumarate-CoA ligase; CHS, chalcone synthase; CHI, chalcone isomerase; 1,2-Rhat, 1,2-rhamnosyltransferase; F3′H, flavonoid 3′-monooxygenase; F3H, flavanone 3-dioxygenase; FLS, flavonol synthase; DFR, dihydroflavonol 4-reductase; ANS, anthocyanidin synthase; LAR, leucocyanidin reductase; UFGT, UDP-glucose flavonoid 3-O-glucosyltransferase; UGT, UDP-glycosyltransferase; 1,6-Rhat, 1,6-rhamnosyltransferase; 7-Glct, flavanone 7-O-glucosyltransferase; 3-Glct, anthocyanidin 3-O-glucosyltransferase; FNS, flavone synthase; F3′5′H, flavanoid 3′,5′-hydroxylase; OMT, O-methyltransferase.

In order to clarify the molecular basis of the differences in flavonoid and lipid contents between HP and RA fruits, we further detected the expression of genes related to flavonoid and lipid metabolism by transcriptional sequencing. Transcriptomic data from the peel and flesh of the two kumquat fruits at three developmental stages showed that a total of 65 structural genes involved in flavonoid biosynthesis showed differential expression between HP and RA fruits. These genes included *PAL*, *C4H*, *4CL*, *CHS*, *CHI*, *F3H*, *F3’H*, *FLS*, *1,2-Rhat*, *UGT*, *DFR*, *ANS*, *UFGT*, and *LAR.* Specifically, the genes that related to early enzymatic reactions during phenylpropanoid pathway, including four *PAL* genes (sjg171720, sjg252410, sjg141140, and sjg170590), three *C4H* genes (sjg256270, sjg023860, and sjg303290), four *4CL* genes (sjg203960, sjg116210, sjg021810, and sjg114460), three *CHS* genes (sjg294150, sjg125680, and sjg125520), and one *CHI* gene (sjg280460), were generally upregulated in the peel of HP during fruit development, particularly at 90 DAF. In contrast, most of the above structural genes demonstrated low expression levels and were downregulated in the flesh of HP compared with RA fruit (Figure 5). Furthermore, nine genes encoding FLS also changed greatly, with four members (sjg143160, sjg125710, sjg143720, and sjg034020) showing upregulation in the peel, while all members showing downregulation in the flesh of HP compared with RA. In addition, six genes related to the anthocyanin biosynthesis pathway were identified and all of them exhibited downregulated expressions in both peel and flesh in HP fruit, with an exception of one *LAR* gene (sjg029150), which was specifically upregulated in HP peel and flesh at 90 DAF. Meanwhile, one *F3H* gene (sjg320640) was continuously downregulated in the peel and flesh, and one *F3’H* gene (sjg186480) was also downregulated in the flesh but upregulated in the peel. We also found that two *1,2-Rhat* genes were regulated, with one (sjg050250) being specifically upregulated and the other (sjg147470) downregulated in the peel, while both downregulated in the flesh (Figure 5). The UGTs catalyze the synthesis of galactosylated derivatives with various substrates, which is a key modifying enzyme in flavonoid metabolism. Here, a total of 24 *UGT* genes were identified and most of them were upregulated in both peel and flesh in HP fruit at all three developmental stages, particularly at 90 and 150 DAF (Figure 5). However, some exceptions were also observed. For instance, one *UGT* member (sjg237020) was continuously downregulated in both HP peel and flesh. In summary, a large number of genes involved in the flavonoid pathway were upregulated in the peel but downregulated in the flesh of HP fruit, which was consistent with the change of flavonoid content in different tissues of the mutant.

**FIGURE 6:**
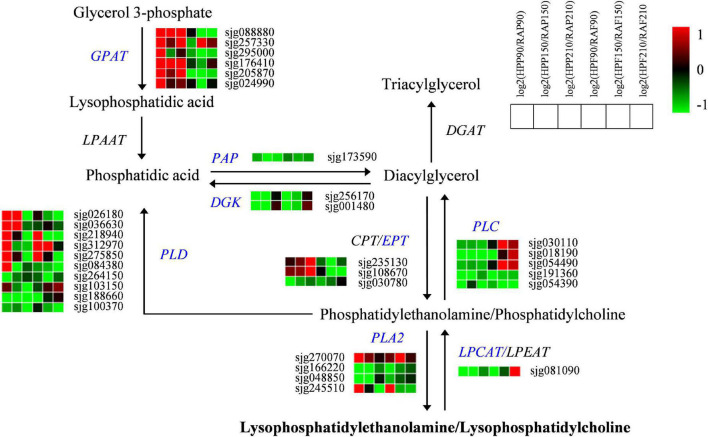
Expression profiles of genes from the glycerophospholipid pathway in the peel and flesh of kumquat fruits. The color scale from green (low) to red (high) represents the values of the log2 ratio (HP/RA) at 90, 150, and 210 DAF. The differentially expressed genes are highlighted in blue. GPAT, glycerol-3-phosphate acyltransferase; LPAAT, Lysophosphatidic acid acyltransferase; PAP, phosphatidate phosphatase; DGK, diacylglycerol kinase; EPT, ethanolaminephosphotransferase; CPT, cholinephosphotransferase; PLA, phospholipase A; PLC, phospholipase C; PLD, phospholipase D; LPCAT, lysophosphatidylcholine acyltransferase; LPEAT, lysophosphatidylethanolamine acyltransferase; DGAT, diacylglycerol O-acyltransferase.

A total of 32 genes associated with glycerophospholipid metabolism were also screened out in the two kumquat fruits (Figure 6). Of these, six genes encoding GPAT were upregulated in the peel, while they were mostly downregulated in the flesh of HP compared with RA kumquat. The expressions of two *LPAAT* members were slightly downregulated but no significant difference was observed between the two kumquat fruits. The activities of PAP and DGK enzymes determined the interconversion of phosphatidic acid and diacylglycerol (DAG). There were one *PAP* gene (sjg173590) and two *DGK* genes (sjg256170 and sjg001480) showing downregulation in the peel and flesh of HP kumquat. The phosphotransferase CPT and EPT can catalyze DAG into phosphatidylcholine (PC) and phosphatidylethanolamine (PE), respectively. Here, three *EPT* genes were identified, namely, two (sjg235130 and sjg108670) upregulated and one (sjg030780) downregulated member in the peel of RA fruit. In the flesh, however, all three *EPT* members were downregulated (Figure 6). We also screened four *PLA2* genes, namely, two downregulated members (sjg166220 and sjg048850), one upregulated member (sjg270070), and one complex expression member (sjg245510) in the peel and flesh. However, all five *PLC* genes were downregulated in HP peel compared with those in RA peel. In the flesh, three of these *PCL* genes (sjg030110, sjg018190, and sjg054490) were upregulated at 150 and 210 DAF. Regarding *PLD* genes, ten members were identified and their expression levels varied depending on developmental stage and fruit tissue. Of these, six *PLD* members (sjg026180, sjg036630, sjg218940, sjg312970, sjg275850, and sjg084380) displayed upregulated expression in the peel, and three members (sjg218940, sjg312970, and sjg275850) displayed upregulated expression in the flesh of HP at 90 and/or 150 DAF. Furthermore, one *LPCAT* gene was downregulated over most of the developmental stages but was upregulated in the flesh of HP fruit at 210 DAF (Figure 6).

## Discussion

The kumquat fruits are widely consumed fresh and have also been used in folk medicine in China (Tan et al., 2016). HP kumquat fruit has drawn increasing attention for its desirable traits, such as glossy peel. Given that the flavonoids play essential roles in determining citrus coloration, we measured the total flavonoid concentration and found it was over four times higher in HP than in RA fruit. Besides, the HP fruit peel was glossier and had less oil gland (Figure 1). Previous reports demonstrated that oil glands in the citrus peel are closely associated with the synthesis and accumulation of important secondary metabolites, such as monoterpenoids and sesquiterpenoids (Liu et al., 2019). Therefore, to further determine metabolite components and understand the morphological differences between the HP and its wild type, widely targeted metabolomics was used to identify the metabolites existing in the peel and flesh of kumquat fruits at the maturation stage. In total, 538 metabolites were identified in the two kumquat varieties. These metabolites were categorized into 17 substances in the secondary classification (Figure 2C and Supplementary Table 1). Among them, flavonoids and lipids were the top two differential metabolite classifications. Besides, hydroxycinnamoyl derivatives are the main differential secondary metabolites between the two kumquat varieties. For example, resveratrol and p-coumaraldehyde were highly expressed in RA fruit, but could not be detected in HP fruit (Supplementary Table 1). Taken together, our results suggested that the HP fruit exhibited a distinct pattern in metabolites accumulation, especially in flavonoids and lipids, and it could be a unique material for germplasm construction and citrus breeding.

### Regulation of Flavonoid Metabolism

Flavonoids constitute a highly diverse family of secondary metabolites and contain about 8,000 known chemicals, which are generally responsible for the color change and flavor development in fruits (Winkel-Shirley, 2001; Ververidis et al., 2007; Khan et al., 2014). Our metabolomic analysis revealed that flavonoids were mostly upregulated in HP peel (Figure 3C), which was consistent with the result of total flavonoid determination (Figure 1B). Flavonoids and carotenoids are the two main pigments in citrus fruits. In general, carotenoids relate to various colors, whereas most flavonoids are colorless except for anthocyanins, which produce “blood” color in blood oranges (Ververidis et al., 2007; Khan et al., 2014). Thus, we conclude that the high flavonoid accumulation in HP peel may contribute to its lighter color when compared with RA fruits. In the flesh, however, differentially accumulated flavonoids were mostly downregulated in HP, with little members in common with the peel. These results supported the finding that flavonoids composition in different tissues of citrus fruits showed a large magnitude variation among the flavedo, albedo, and juice vesicle tissues (Zhang et al., 2011). In addition, the compositions and quantities of flavonoids in kumquat fruit are rather different from other citrus species. It is reported that *O*-glycosylated flavonoids are rich in all fruit tissues from *Citrus* genus (Wang et al., 2017), whereas *C*-glycoside flavonoids are dominant phenolic compounds in kumquat fruit (Lou and Ho, 2017). However, herein metabolomic analysis uncovered that flavonoids are mostly in the form of *O*-glycosides in HP and RA kumquat fruits. In addition, the flavedo of *Citrus* genus widely contains PMFs, another kind of special flavonoid subclass, which have high oral bioavailability, antioxidant, and antibacterial activities (Zhang et al., 2012). In HP peel, several PMFs such as nobiletin (Cit305) and tangeretin (Cit367), which can cause lingering bitterness of citrus fruits, were identified and showed downregulation (Figure 4A). Another bitter substance, limonin, was also highly expressed but accumulated lower in the peel of HP than in the peel of RA (Supplementary Table 1). The low accumulation of these compounds could reduce the medicinal value of HP fruit but is an important reason for its pleasant taste.

Flavonoid biosynthesis is primarily regulated by phenylalanine, which is catalyzed by a series of genes involving the phenylpropanoid pathway, such as *PAL*, *C4H*, and *4CL*, and provides precursors for major phenolic metabolites biosynthesis in higher plants (Winkel-Shirley, 2001). Chen et al. (2021) reported that *PAL* and *4CL* are important genes in flavonoids accumulation in the yellow peel of cucumber. Our results suggested that at least four *PAL* and *4CL* genes and three *C4L* genes may be involved in the early reactions of flavonoids biosynthesis in HP peel. However, those genes showed downregulation during HP flesh development. In addition, *CHS* and *CHI* are also two key enzyme genes of intermediate flavonoid biosynthesis in plants. Previous studies reported that the expression level of *CHS* and *CHI* genes is closely associated with the content of flavonoids in citrus fruits (Moriguchi et al., 2001; Wang et al., 2010). Functional characterization of both genes was also conducted by Dong et al. (2001), who introduced *CHS* (*C2*) and *CHI* (*CHI1*) genes of maize into Arabidopsis mutants and found that the transgenic plants could accumulate flavonoids and anthocyanins. Similar results were obtained when a *Freesia hybrida CHS* gene (*FhCHS1*) was transformed into Arabidopsis *tt4* mutants (Sun et al., 2015). The upregulated expression of *CHS* and *CHI* members in HP peel was supported by their potential functions in the biosynthesis of anthocyanins and flavonoids (Tian et al., 2020).

Dihydroflavonols are kind of branching-point metabolites, which can be converted either by *DFR* in the first step for anthocyanin biosynthesis or by *FLS* to produce various flavonols and glycosidic derivatives (Erlund, 2004; Khan et al., 2014). Therefore, the downregulation of *DFR*, as well as other related genes including *ANS*, *LAR*, and *UFGT*, could limit anthocyanin synthesis in HP fruit. Meanwhile, the upregulation of *FLS* genes suggested their positive roles in flavonol accumulation in HP, particularly in the peel. Our results also implied that there is a competitive relationship between flavonol and anthocyanin biosynthesis in HP kumquat like in Arabidopsis (Gou et al., 2011), but different with tea plant, in which flavonoids were accompanied by anthocyanin (Shen et al., 2018). The HP fruit mainly accumulated *O*-glycosylated flavonoids, which are generated by activities of various UGTs (Osmani et al., 2010). At present, *UGTs* have been reported in many plant species (Caputi et al., 2012; Kumar et al., 2013). Recently, a *UGT* member from sweet orange (*CsUGT76F1*) was isolated, and its functions as flavonoid 7-O-glucosyltransferase and 7-O-rhamnosyltransferase were also established *in vivo* (Liu et al., 2018). As indicated in the present study, we screened out many *UGTs* that may contribute to glycosylated flavonoid accumulation in kumquat. Among them, three members (sjg158400, sjg283140, and sjg299230) increased their transcript abundance in all tested samples of HP fruit and likely played significant roles in flavonoid glycosylation (Figure 5). Further functional characterizations of these *UGTs* are essential for exploring their roles in the modification of biological activities and accumulation of flavonoids in the kumquat fruits.

### Regulation of Lipid Metabolism

Lipid compounds are mainly categorized into eight subgroups, namely, fatty acyls, glycerolipids, polyketides, sphingolipids, prenol lipids, sterol lipids, saccharolipids, and glycerophospholipids (Liebisch et al., 2020). The citrus fruit is mostly composed of lipids belonging to fatty acids and glycerophospholipids (Kolesnik et al., 1989; Touns et al., 2011). Recently, studies on the glossy surface of the ‘Newhall’ navel orange mutant demonstrated that the decrease in aliphatic wax components such as aldehydes, alkanes, alcohols, and fatty acids was related to its glossy phenotype (Liu et al., 2015; He et al., 2019). Another study found that the plastid lipids (including monogalactosyldiacylglycerols, digalactosyldiacylglycerols, and lysophosphatidylglycerols) and the phospholipid precursors (including phosphatidic acids and DAGs) were highly accumulated in the glossy mutant (Wan et al., 2020). Although HP fruit possessed glossy peel, it showed a decrease in the lipids, particularly the LysoPCs and LysoPEs in the peel and flesh, compared with those in RA fruit. This could be partly ascribed to the few amount of oil glands observed in HP peel (Figure 1E). Previous studies reported that lipids are components or precursors of essential oils, which are mostly synthesized and accumulated in oil gland in citrus fruits (Voo et al., 2012; Liu et al., 2019). Lysophospholipids such as LysoPEs and LysoPCs are present only in trace amounts but they are involved in cell expansion, wound response, and freezing acclimation in plants (Cowan, 2006). Furthermore, several reports provide support for the roles of lysophospholipids in retarding leaf senescence and enhancing fruit shelf life (Hong et al., 2009). Thus, it is of great importance to investigate whether the decrease in LysoPCs and LysoPEs reduces the storage quality of HP fruit.

Lysophosphatidycholines and LysoPEs can be hydrolyzed from PCs and PEs, which are synthesized from glycerol 3-phosphate by the activation of a series of enzymes such as GPAT, LPAAT, PAP, DGK, CPT, and EPT (Cowan, 2006; Nakamura, 2017). Xu et al. (2021) reported that glycerophospholipid metabolism is responsible for the postharvest softening of pears, and the expressions of *GPAT1* (glycerol-3-phosphate acyltransferase), *CCT2* (cholinephosphate cytidylyltransferase 1), *LCAT3* (phospholipase A), *LCAT4* (lecithin-cholesterol acyltransferase), and *NPC1* (non-specific phospholipase C1) are significantly correlated with the contents of PE and PC. In the present study, different numbers of *GPAT*, *PAP*, *DGK*, and *EPT* genes were observed to be differentially regulated between HP and RA fruits, indicating their important roles in the glycerophospholipid metabolism of these two kumquats. The plant phospholipases hydrolyzed glycerophospholipids at different ester bonds. They can be grouped into phospholipase A (PLA1 and PLA2), phospholipase C, and phospholipase D depending on the cleaved site in the phospholipid molecule (Filkin et al., 2020). PLA2 hydrolyzes the sn-2 position of glycerophosphates and produces lysophospholipids (Nakamura, 2017), while lysophospholipid: acyl-COA acyltransferases (LPCAT/LPEAT) can acylate lysophospholipids and reduce its level (Jasieniecka-Gazarkiewicz et al., 2017). Thus, the downregulation of two *PLA2* and upregulation of one *LPCAT* in HP could be associated with the low accumulations of lysophospholipids, including LysoPEs and LysoPCs in the fruits. In addition to PLA, both PLC and PLD can hydrolyze the glycerophosphates, resulting in the generation of DAG and phosphatidic acid, respectively (Filkin et al., 2020). Therefore, the upregulation of *PLC* and *PLD* may accelerate the degradation of glycerophospholipid and limit supply for lysophospholipids synthesis and finally, responsible for the low concentrations of LysoPEs and LysoPCs in HP fruit. Collectively, the differences in lysophospholipid accumulations and related gene expressions suggested a possible change of the glycerophospholipid pathway in HP variety.

## Conclusion

HP fruit displayed integrative differences in morphological and physiological features compared with RA fruit. These observed differences may be ascribed to the differential accumulation of metabolites, particularly the high glycosylated flavonoid and low lysophospholipid compounds in HP fruit. Furthermore, various genes involved in flavonoid and lipid metabolisms were differentially expressed. The upregulations of *PAL*, *C4H*, *4CL*, *CHS*, and particularly the *UGT* genes contributed to high flavonoid accumulations in HP fruit. On the other hand, expression levels of the lipid degradation-related genes, including *LPCAT*, *PLC*, and *PLD*, were also upregulated, resulting in low lysophospholipid accumulations in HP fruit. Together, our results reveal that the flavonoid and lipid pathways are regulated at the transcriptional levels, which provide comprehensive insights into the regulatory network of both components in kumquat fruits.

## Data Availability Statement

The datasets presented in this study can be found in online repositories. The names of the repository/repositories and accession number(s) can be found in the article/Supplementary Material.

## Author Contributions

QW and QM designed the experiment. YH and XD performed the study. QW, QG, and QM drafted the manuscript. GZ and XL revised the manuscript. All authors read and approved the final version of this manuscript.

## Conflict of Interest

The authors declare that the research was conducted in the absence of any commercial or financial relationships that could be construed as a potential conflict of interest.

## Publisher’s Note

All claims expressed in this article are solely those of the authors and do not necessarily represent those of their affiliated organizations, or those of the publisher, the editors and the reviewers. Any product that may be evaluated in this article, or claim that may be made by its manufacturer, is not guaranteed or endorsed by the publisher.
