# Supplementary material for: Metabolic Profiling and Gene Expression Analysis Unveil Differences in Flavonoid and Lipid Metabolisms Between ‘Huapi’ Kumquat (Fortunella crassifolia Swingle) and Its Wild Type
Source: Front Plant Sci. 2021 Dec 2;12:759968. doi: 10.3389/fpls.2021.759968 (PMC8675212; doi:10.3389/fpls.2021.759968)
Supplement: Supplementary file 1 [file Data_Sheet_1.PDF]

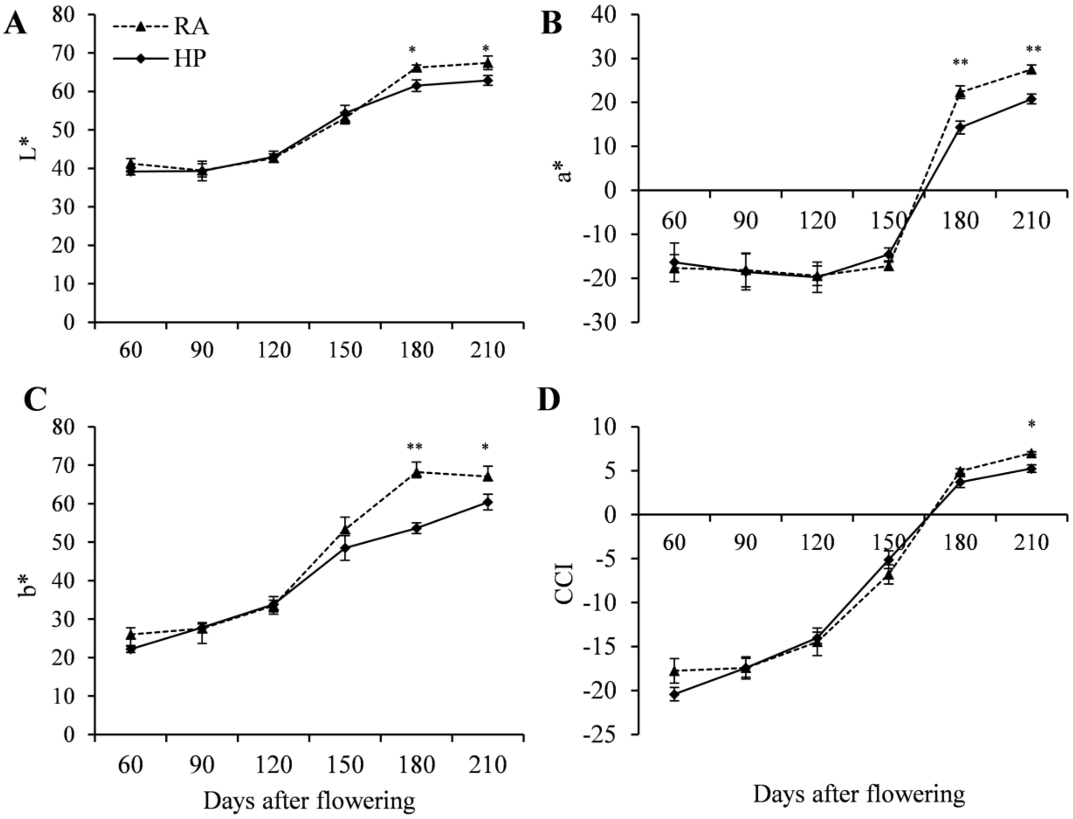

Supplementary Figure S1. Dynamic changes in color indices including **(A)** L^*, **(B)** a^*, **(C)** b^* and **(D)** CCI values of RA and HP kumquat varieties during fruit development. Asterisk indicate the difference between verities at the same development stage (**P* < 0.05 and ***P* < 0.01).

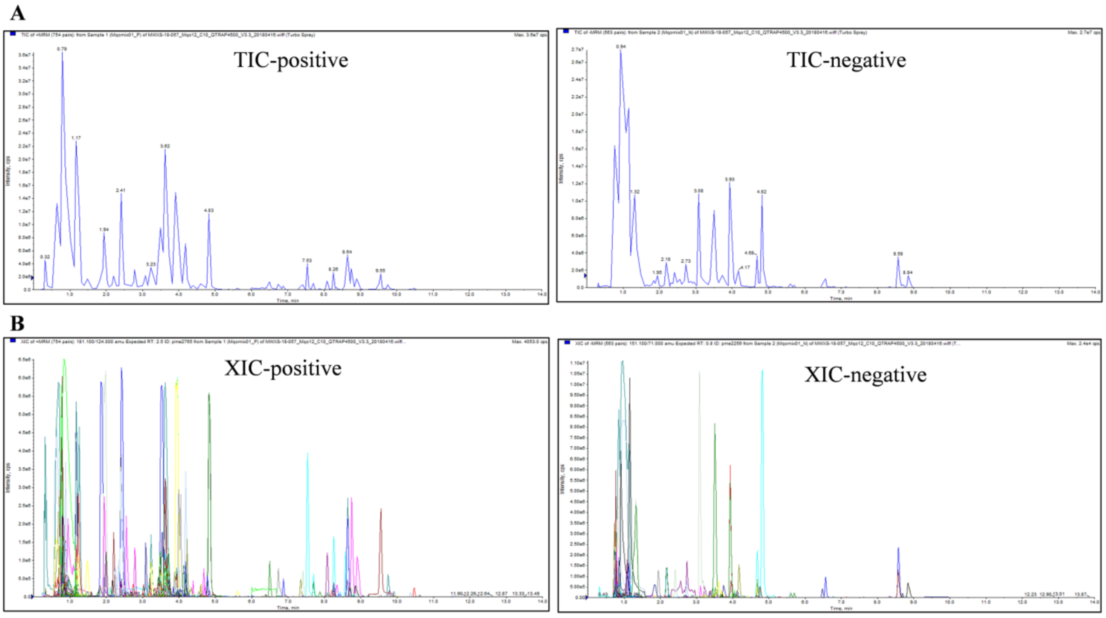

Supplementary Figure S2. Qualitative and quantitative analysis of metabolites. **(A)** Total ions current and **(B)** MRM metabolite detection multi-peak diagram. X and Y axis are retention time and total intensity, respectively.

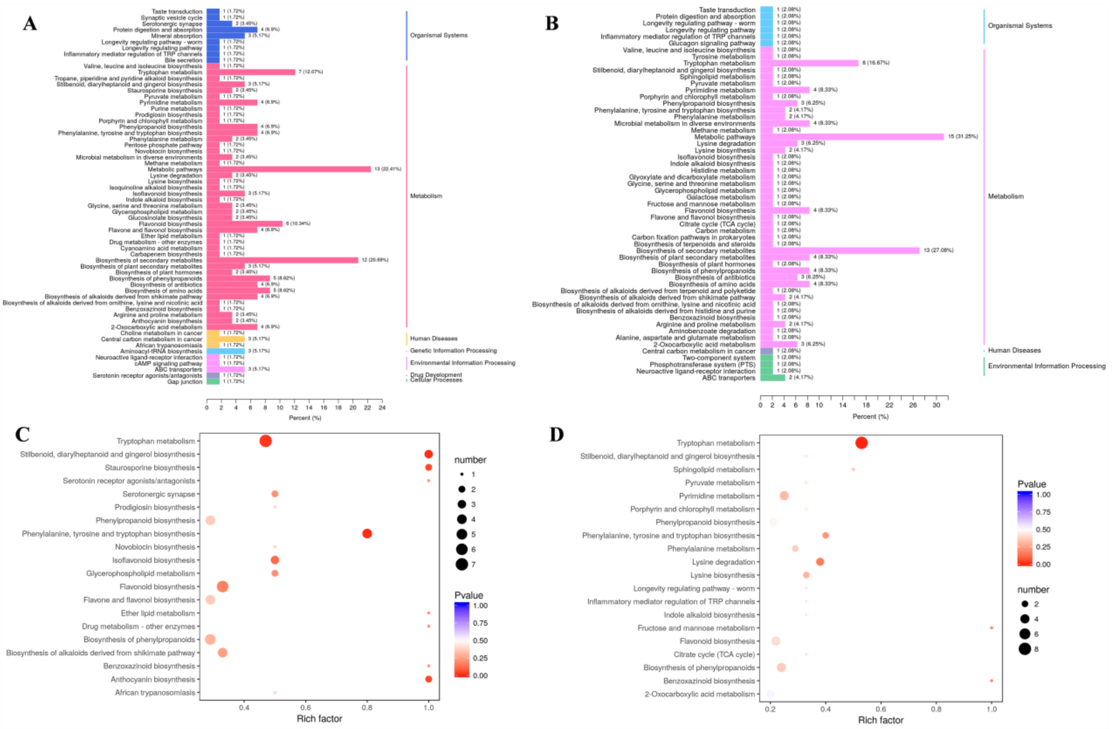

Supplementary Figure S3. KEGG analysis of the differentially accumulated metabolites. Classification analysis of metabolites **(A)** in the peel and **(B)** in the flesh. Functional enrichment analysis of metabolites **(C)** in the peel and **(D)** in the flesh.
